# Monoamines as Drug Targets in Chronic Pain: Focusing on Neuropathic Pain

**DOI:** 10.3389/fnins.2019.01268

**Published:** 2019-11-26

**Authors:** Lidia Bravo, Meritxell Llorca-Torralba, Esther Berrocoso, Juan Antonio Micó

**Affiliations:** ^1^Neuropsychopharmacology and Psychobiology Research Group, Department of Neuroscience, University of Cádiz, Cádiz, Spain; ^2^Instituto de Investigación e Innovación Biomédica de Cádiz, INiBICA, Hospital Universitario Puerta del Mar, Cádiz, Spain; ^3^Centro de Investigación Biomédica en Red de Salud Mental (CIBERSAM), Instituto de Salud Carlos III, Madrid, Spain; ^4^Neuropsychopharmacology and Psychobiology Research Group, Department of Psychology, University of Cádiz, Cádiz, Spain

**Keywords:** chronic pain, neuropathic pain, monoamines, antidepressants, noradrenaline, serotonin

## Abstract

Monoamines are involved in regulating the endogenous pain system and indeed, peripheral and central monoaminergic dysfunction has been demonstrated in certain types of pain, particularly in neuropathic pain. Accordingly, drugs that modulate the monaminergic system and that were originally designed to treat depression are now considered to be first line treatments for certain types of neuropathic pain (e.g., serotonin and noradrenaline (and also dopamine) reuptake inhibitors). The analgesia induced by these drugs seems to be mediated by inhibiting the reuptake of these monoamines, thereby reinforcing the descending inhibitory pain pathways. Hence, it is of particular interest to study the monoaminergic mechanisms involved in the development and maintenance of chronic pain. Other analgesic drugs may also be used in combination with monoamines to facilitate descending pain inhibition (e.g., gabapentinoids and opioids) and such combinations are often also used to alleviate certain types of chronic pain. By contrast, while NSAIDs are thought to influence the monoaminergic system, they just produce consistent analgesia in inflammatory pain. Thus, in this review we will provide preclinical and clinical evidence of the role of monoamines in the modulation of chronic pain, reviewing how this system is implicated in the analgesic mechanism of action of antidepressants, gabapentinoids, atypical opioids, NSAIDs and histaminergic drugs.

## Introduction

The monoaminergic system is implicated in the maintenance of homeostasis in the nervous system and among their distinct functions, monoamines regulate the endogenous pain system (Bannister et al., 2009). In terms of chronic pain, some recent contributions to the pharmacological arsenal of analgesics that target monoamines are selective inhibitors of serotonin and noradrenaline reuptake (SNRIs, e.g., duloxetine), and atypical opioids that combine their opioid activities with an effect on monoamines (e.g., tramadol and tapentadol). These drugs have opened the way to consider monoamines and their receptors as potential targets to develop innovative analgesics. Moreover, these drugs have also helped clarify the mechanism of action of some so-called atypical analgesics, the primary effects of which do not involve the monoaminergic system (i.e., tramadol, tapentadol, nefopam) even though their global efficacy as analgesics might well be driven by monoamines.

The most classic drugs that act directly on the monoaminergic system are antidepressants, drugs that were not originally designed as analgesics but that are currently considered first line analgesics for some types of neuropathic pain (Finnerup et al., 2005; Sindrup et al., 2005). Nevertheless, not all antidepressants have analgesic properties. For years, amitriptyline has been the most widely prescribed antidepressant but more recently, the SNRI duloxetine has been positioned as an analgesic antidepressant that can be used to treat peripheral diabetic neuropathic pain and fibromyalgia. By contrast, selective serotonin reuptake inhibitors (SSRIs) are not suitable analgesics to treat neuropathic pain. Atypical opioid drugs combine an opioid mechanism of action with another monoaminergic one, producing effective analgesia in several types of chronic pain, not only neuropathic pain (e.g., tramadol and tapentadol: Bravo et al., 2017; Coluzzi et al., 2017). In this review, we will present information from various preclinical and clinical studies that investigated the role of monoamines in chronic pain, as well as studies that assessed the mechanisms of action of actual and potential analgesics to treat different types of chronic pain.

## Types of Pain

Pain is necessary for our survival as it is a process that helps protect us from danger and it serves as a short-term response to resolve injury. However, pain that persists for longer than 3 months is considered to be a pathological state known as chronic pain. Based on its etiology, pain is classified as: (i) nociceptive, caused by lesion or potential tissue damage that directly stimulates C and Aδ fibers (e.g., somatic and visceral pain); (ii) inflammatory, caused by an inflammatory process (e.g., arthritis); (iii) neuropathic, caused by a lesion or disease affecting the somatosensory system (e.g., diabetic neuropathy, post-herpetic and neuralgic sciatic nerve injury). These three main types of pain can develop into chronic pain, although some particular types of chronic pain may not be properly covered by the categories of “nociceptive” or “neuropathic” pain, and they can be categorized as “nociplastic pain.” This fourth type of pain is characterized by altered nociception in the absence of clear evidence of tissue damage or lesion, or of disease that affects the somatosensory system (*International Association for Study of Pain*, https://www.iasp-pain.org/PublicationsNews/NewsDetail.aspx?ItemNumber=6862).

Regardless of its etiology, chronic pain is a public health problem that affects 20% of the adult population in Europe (Van Hecke et al., 2013). Chronic pain is one of the most frequent reasons for visits to a physician and its estimated financial cost exceeds €200 billion per annum in Europe and $150 billion per annum in the United States (Tracey and Bushnell, 2009). One of the main problems of chronic pain is its inadequate management, most likely caused by ignorance of its biological roots. The monoaminergic system can modulate pain signaling and this system undergoes important alterations under conditions of chronic pain, contributing to its persistance. In fact, many types of chronic pain are now treated with medications that affect monoamines and as such, these will be discussed exhaustively in the present review.

## Pain Modulation by Monoamines

The monoaminergic system is an endogenous modulator of pain (Bannister and Dickenson, 2016). Peripheral nociceptive signals reach the spinal cord and the pain messages are relayed by ascending projections targeting the thalamus, the dorsal reticular nucleus (DRt), the rostral ventromedial medulla (RVM), and the midbrain periaqueductal gray (PAG) which integrates forebrain influences. The rostral projections from the thalamus target areas that several sub-regions in the cortex and the amygdala where both the sensory and affective components of pain are processed. In parallel, descending monoaminergic pain pathways, mainly from the Locus Coeruleus (LC) and RVM, modulate the ascending nociceptive information at the level of the spinal cord. These modulatory effects are mainly mediated by serotonin (5-HT) and noradrenaline (NA), which may well represent potential targets for new analgesic drugs (see below).

### Noradrenergic Pain Modulation

The noradrenergic system is mainly implicated in descending inhibitory pain with the noradrenergic descending projections to the spinal cord mainly arising from the A6 (LC), A5 and A7 (Howorth et al., 2009). The effects of NA are mediated by the α and β-NA adrenergic receptors, which are expressed widely in the central and peripheral nervous systems (CNS and PNS), with particularly strong expression in areas that directly participate in pain processing (e.g., PAG, RVM, thalamus, LC, prefrontal cortex and amygdala). There are various subtypes of adrenoceptors (ARs), a- and β-ARs (α1A, α1B, α1D, α2A, α2B, and α2C; and β1, β2, and β3: Ruffolo and Hieble, 1994), with the α2-ARs most commonly associated with pain modulation. As we will describe here, these receptors may be suitable pharmacological targets to treat chronic pain.

#### Peripheral Noradrenergic Modulation

Noradrenaline is released locally by post-ganglionic sympathetic nerve fibers (Pertovaara, 2006). Many α-ARs subtypes have been identified in the dorsal root ganglia (DRGs: α1A, α1B, α1D, α2A, and α2C) and also on the nerve fibers distributed to the skin (Dawson et al., 2011) where peripheral nociception is locally modulated (Shi et al., 2000; Xie et al., 2001; Maruo et al., 2006). Although α2-AR agonists mainly produce analgesia via spinal and supraspinal action (Bernard et al., 1994; Buerkle and Yaksh, 1998; Asano et al., 2000), peripheral ARs also fulfill an important role in analgesia (Pertovaara, 2006). Thus, topical α2 agonist administration produces analgesia, an effect that is blocked by pretreatment with AR antagonists in acute and chronic pain rat models (Nakamura and Ferreira, 1988; Dogrul and Uzbay, 2004). Accordingly, topic administration of clonidine relieves hyperalgesia in patients with complex regional pain syndrome (Davis et al., 1991), indicating that peripheral stimulation of α2-ARs could relieve neuropathic pain. β2-ARs are located on peripheral terminals and cell bodies of primary afferent nociceptors (Aley et al., 2001). Nevertheless, it remains unclear if activation of β2-ARs is pro- or anti-nociceptive. Most preclinical studies have shown that peripheral β2-AR stimulation by antidepressants induces anti-allodynic effects in neuropathic pain models (Bohren et al., 2013; Kremer et al., 2018), while intradermal injection of epinephrine produces hypersensitivity (Khasar et al., 1999; Parada et al., 2003).

#### Central Noradrenergic Modulation

Preclinical studies reported that chemical or electrical stimulation of the LC, the main noradrenergic nucleus, induces analgesia via the spinal cord that is blocked by spinal α2-AR antagonists (Pertovaara, 2006). The LC sends projections to all cortical regions, as well as to the basolateral amygdala (BLA), hippocampus, and ventral tegmental area (VTA), all of which express adrenergic receptors and are relevant structures in the context of pain (Loughlin et al., 1986; Sara and Bouret, 2012). Moreover, the LC express a large proportion of the α2-AR inhibitory auto-receptors that modulate NA release via these projections (Llorca-Torralba et al., 2016). Despite the prevalence of studies focusing on the LC in pain modulation, there is also evidence that other noradrenergic nuclei play a role in pain processing (Llorca-Torralba et al., 2016). Indeed, the A5 and A7 noradrenergic clusters provide significant NA input to the spinal cord, where the final balance between inhibitory and facilitatory modulation of pain takes place (Pertovaara, 2006; Bruinstroop et al., 2012). These findings provide evidence of the complex interaction between NA and pain. At present, the role of NA on spinal adrenergic receptors seems to be clearer than that its effects on supraspinal noradrenergic receptors.

### Serotonergic Pain Modulation

Serotonin (5-HT, 5-hydroxytryptamine) has been widely related to pain modulation through peripheral and central actions. Unlike NA, which action seem to be more related to analgesia, 5-HT acts on specific receptors that contribute to the maintenance of pain (Suzuki et al., 2004; Bannister and Dickenson, 2016).

#### Peripheral Serotonergic Modulation

Serotonergic nociceptive transmission at peripheral sensory nerves is mediated by several subtypes of 5-HT receptors (5-HT1B, 5-HT1D, 5-HT2A, 5-HT2B, 5-HT3, 5-HT4, and 5-HT7) known to exist in DRGs (Pierce et al., 1996; Wu et al., 2001). Numerous studies have shown that peripheral administration of 5-HT increases the excitability of Aδ and C fibers, and DRG neurons (Michaelis and Janig, 1998; Lang et al., 2006), suggesting a pro-nociceptive role for 5-HT. Indeed, intradermal injection of 5-HT in rats produces dose-dependent hypersensitivity mediated by 5-HT1A receptors (Taiwo et al., 1992). Moreover, peripheral injection of 5-HT2A, 5-HT3, and 5-HT7 antagonists produces an anti-nociceptive effect in a model of the formalin pain test (Rocha-González et al., 2005; Nakajima et al., 2009; Godinez-Chaparro et al., 2011). In clinical studies on healthy volunteers, intradermal injection of 5-HT produces burning pain (Lischetzki et al., 2001) and the hypersensitivity induced by 5-HT injections into the masseter muscle is antagonized by a 5-HT3 antagonist (Ernberg et al., 2000).

#### Central Serotonergic Modulation

Descending serotonergic pathways include the RVM, where the midline raphe magnus nucleus (RMN) can inhibit or facilitate descending pain (Fields, 2004). ON and OFF cells, respectively, in the RVM send descending inhibitory and excitatory fibers to the dorsal horn spinal cord neurons that control the spinal sensory transmission (Urban and Gebhart, 1999; Neubert et al., 2004). Thus, activation of the RVM by electrical stimulation or glutamate microinjection evokes the spinal release of 5-HT, inducing analgesia (Oliveras et al., 1975; Satoh et al., 1983; Aimone and Gebhart, 1986). The RVM receives projections from the periaqueductal gray (PAG) and the microinjection of opioids in RVM has an inhibitory effect on noxious stimulation, indirectly activating OFF cells and thereby reducing pain (Zorman et al., 1981; Jensen and Yaksh, 1989; Heinricher et al., 1994).

It was largely demonstrated that the effect of spinal 5-HT is either inhibitory or facilitatory, depending on the 5-HT receptor subtypes activated (Green et al., 2000; Millan, 2002; Dogrul and Uzbay, 2004; Suzuki et al., 2004). In pain modulation, 5-HT exerts excitatory effects via the 5-HT2 and 5-HT3 receptors, yet an inhibitory effect is provoked by stimulation of 5-HT7 receptors (Obata et al., 2001; Viguier et al., 2013; Bannister et al., 2017). However, the 5-HT1A receptor could exert excitatory or inhibitory effects (Millan, 2002). Except to treat chronic migraine, no drugs targeting 5-HT receptors are used for chronic pain. Thus, central action of 5-HT clearly needs further investigation in order to understand how 5-HT modulates pain, and its implication as a possible analgesic agent in acute and chronic pain.

### Dopaminergic Pain Modulation

The role of dopamine (DA) in pain modulation has received less attention than that of NA and 5-HT. The dopaminergic system is widely known to participate in mesocorticolimbic reward system, which arises from the VTA and modulates emotion-related behavior (Baik, 2013). Interestingly, dopamine plays a role in the modulation of nociceptive transmission at both the spinal and supraspinal levels (Potvin et al., 2009). In the CNS, there are five subtypes of DA receptors (D1-D5), and the D1 and D2 receptors are those most strongly implicated in pain modulation, as witnessed in animal models (Millan, 2002). As such, activation of D2 receptors at the spinal level induces an anti-nociceptive effect (Magnusson and Fisher, 2000; Taylor et al., 2003; Ansah et al., 2007; Coffeen et al., 2008; Meyer et al., 2009), whereas stimulation of D1 receptors is pro-nociceptive (Gao et al., 2001; Yang et al., 2005; Kim et al., 2015).

Focal electrical stimulation of descending dopaminergic projections suppresses painful transmission at the spinal cord level, mainly through the A11 nucleus of the pericentral posterior hypothalamus and substantia nigra (Fleetwood-Walker et al., 1988). Thus, both acute and sustained noxious stimuli increase DA release to D2 receptors of spinothalamic and primary nociceptive neurons, eliciting an anti-nociceptive response (Millan, 2002). D1 receptor activation in the PAG or insular cortex has also been seen to attenuate pain-related behavior, presumably through the activation of neurons involved in descending inhibition (Burkey et al., 1999; Flores et al., 2004). The spinal effect of DA appears to depend on its local concentration. Low levels of DA may activate the anti-nociceptive D2 receptors, whereas higher levels would activate the pro-nociceptive D1 receptors (Paulus and Trenkwalder, 2006).

Interestingly, there appears to be an interaction between the noradrenaline transporter (NAT) and DA release in areas like the VTA, LC and dorsal hippocampus (Moron et al., 2002; Carboni and Silvagni, 2004; Guiard et al., 2008). Similarly, a small population of DA-synthesizing cells exists in DRGs (Millan, 2002) and consequently, it is possible that noradrenergic neurons constitute an important source of DA in the dorsal horn to control pain. Moreover, there is also an interaction between the DA and opioid system, and significantly, dopaminergic neurons are necessary for the anti-nociceptive effect of morphine, particularly in the PAG (Flores et al., 2004; Meyer et al., 2009). These findings suggest that dopaminergic pathways and their regulation by noradrenergic inputs fulfill an important role in the control of nociceptive transmission. In this context, antidepressants that act on the dopaminergic system might represent new therapeutic tools in the treatment of chronic pain, such as bupropion or more recently, triple reuptake inhibitors (TRIs: Xie et al., 2001; Hache et al., 2011; Navratilova et al., 2012).

### Histamine in Pain Modulation

Histamine 2-(4-Imidazolyl)ethylamine) is released from neuronal and non-neuronal sources and it is a mediator of many physiological processes, including the modulation of pain. Histamine is released peripherally in response to tissue injury that directly sensitizes nociceptors and it contributes to the generation of pain hypersensitivity (Kajihara et al., 2010). By contrast, the role of histamine in the CNS remains unclear. Preclinical studies have shown that histamine injected directly into supraspinal areas (e.g., the somatosensory cortex or hippocampus) attenuates pain (Erfanparast et al., 2010; Tamaddonfard and Hamzeh-Gooshchi, 2014), while intrathecal injection of histamine appears to facilitate nociception (Yoshida et al., 2005). Histamine interacts with four receptor subtypes (H1, H2, H3 and H4), which are expressed in both presynaptic and post-synaptic neurons (Brown et al., 2001). H1 and H2 are excitatory receptors mainly located postsynaptically (Brown et al., 2001; Zhang et al., 2013), and systemic inhibition of these receptors has antinociceptive effects in rats when assessed with the formalin test (Mojtahedin, 2016). H3 receptors are predominantly expressed in the central and peripheral nervous system (Panula et al., 2015). This receptor is mainly localized presynaptically in histaminergic neurons with inhibitory activity that regulate the levels of histamine (Hough and Rice, 2011), while its post-synaptic localization in non-histaminergic neurons regulates the release of neurotransmitters, such as ACh, dopamine, 5-HT and noradrenaline (Obara et al., 2019). H4 receptors possess inhibitory activity and they are expressed in neurons, although their location in the nervous system remains uncertain (Connelly et al., 2009). Thus, histamine exerts different effects on pain modulation depending on the receptor subtype with which it interacts and no histaminergic agents have been approved until now for the management of chronic pain.

## Monoaminergic Dysfunction in Chronic Pain

**FIGURE 1:**
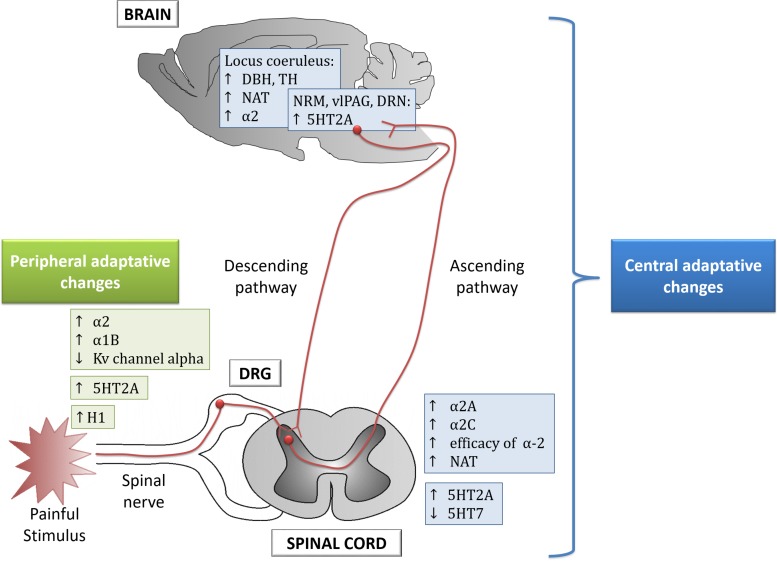
Peripheral and central adaptive changes observed in animal models of chronic pain. DRG, dorsal root ganglion; DRN, dorsal raphe nucleus; RMN, raphe magnus nucleus; vlPAG, ventrolateral periaqueductal gray; DBH, dopamaine beta-hydroxylase; TH, tyrosine hydroxylase; NAT, noradrenaline transporter.

Chronic pain induces monoaminergic plasticity at peripheral and central areas which lead to the classical signs of persistent pain (Figure 1 and Table 1; Hains et al., 2002; Sounvoravong et al., 2004; Heinricher et al., 2009; Morgado et al., 2011; Alba-Delgado et al., 2013). Thus, a review of the monoaminergic dysfunction in chronic pain may identify potential drug targets.

**TABLE 1:** Monoaminergic dysfunction in preclinical models of pain.

**System**	**Finding reported**	**Model of pain**	**Animal**	**References**
**Noradrenergic**				
*Peripheral*	↑α2-AR	Sciatic nerve ligation	Rat	McLachlan et al., 1993
(DRG)	↑α1B-AR	SNL, Sciatic nerve ligation	Rat	Xie et al., 2001; Maruo et al., 2006
	↓ Kv channel alpha	CCI	Rat	Kim et al., 2001, 2002
*Spinal*	↑α2A-AR	Inflammatory pain	Sheep	Brandt and Livingston, 1990
	↑α2C-AR	SNL	Rat	Stone et al., 1999
	↑ Efficacy of coupling between α-2AR and Gα	SNL	Rat	Bantel et al., 2005
	↑ NAT	SNL	Rat	Rojo et al., 2012
*Supraspinal*	↑ TH			
(locus coeruleus)	↑ NAT	CCI	Rat	Alba-Delgado et al., 2013
	↑α2-AR			
	↑ DBH	SNL	Rat	Tazawa et al., 2015
**Serotonergic**				
*Peripheral*	↑ 5-HT2A in DRG	Vincristine-induced neuropathy	Rat	Thibault et al., 2008
*Spinal*	↑ 5-HT2A in ipsilateral side of dorsal horn	CFA	Rat	Zhang et al., 2001, 2002
	↑ 5-HT2A in the lumbar dorsal horn	Vincristine-induced neuropathy	Rat	Thibault et al., 2008
	↓ 5-HT7 in dorsal spinal cord	Formalin test	Rat	Rocha-González et al., 2005
	↓ 5-HT7 in ipsilateral dorsal spinal cord	SNL	Rat	Amaya-Castellanos et al., 2011
*Supraspinal*	↑ 5-HT2A bilateral NRM	CFA	Rat	
	↑ 5-HT2A vlPAG	CFA	Rat	Zhang et al., 2001, 2002
	↑ 5-HT2A DRN	CFA	Rat	
**Dopaminergic**				
*Supraspinal*	↓ D2 receptor in the NAc	SNI	Rat	Sagheddu et al., 2015
	Disruption on D2 and D3 receptors in the hippocampus	SNI	Rat	Cardoso-Cruz et al., 2014
**Histaminergic**				
*Peripheral*	↑ H1 receptor in nociceptive afferent neurons	Capsaicin-induced hyperalgesia	Guinea pig	Kashiba et al., 2001

*AR, adrenoceptor; CCI, chronic constriction injury; CFA, complete Freund’s adjuvant; DBH, dopamine beta hydroxylase; DRG, dorsal root ganglion; DRN, dorsal raphe nucleus; NAc: accumbens nucleus; NAT, noradrenaline transporter; NRM: raphe magnus; SNI, spared nerve injury; SNL, spinal nerve ligation; TH, tyrosine hydroxylase; vlPAG, ventrolateral periaqueductal gray.*

### Noradrenaline

Peripheral α-adrenergic stimulation does not affect pain sensation in healthy conditions but interestingly, such excitation enhances pain sensation in chronic pain (Shea and Perl, 1985). This pro-nociceptive effect is the result of increased excitability of primary sensory neurons, known as hypersensitivity to innocuous and noxious stimuli. The increase in excitability is the result, in part, of neuroplastic changes in animal models of chronic pain, driven possibly by an increase in α2- and α1B-AR mRNA expression in DRGs (Sato and Perl, 1991; McLachlan et al., 1993; Bossut and Perl, 1995; Xie et al., 2001; Maruo et al., 2006) and by upregulation of α1-ARs on cutaneous nociceptive afferents (Drummond et al., 2014). Interestingly, this excitability is enhanced by α2 agonists while it is blocked by administration of α2 antagonists (Sato and Perl, 1991; McLachlan et al., 1993). Based on these studies, it was proposed that the effect of the increase in α2-AR expression on afferents contributes to the maintenance of chronic pain, making this a potential target to relieve peripheral chronic pain.

Spinal α-ARs are thought to play an opposite role to peripheral ARs in chronic pain. An increase in spinal α2A-ARs has been reported in sheep with inflammatory pain (Brandt and Livingston, 1990), while in rats, neuropathic pain has been associated with an increase in spinal α2C-ARs but not α2A-ARs (Stone et al., 1999). Similarly, the number of spinal α2A-ARs in spinal nerve ligation (SNL) rats remains unaltered but surprisingly, an increase in the efficacy of coupling between α2A and Gα subunits has been reported (Bantel et al., 2005). Additionally, upregulation of the spinal NAT has been reported in such SNL rats (Rojo et al., 2012). These results suggest that chronic pain induces adaptive changes in response to reduced descending noradrenergic tone (Hughes et al., 2013). Interestingly, the increase in α2A-AR efficiency could explain why antidepressants induce spinal analgesia in chronic pain conditions (Luo et al., 1994; Malmberg et al., 2001; Bantel et al., 2005). These central adaptive changes suggest that after nerve injury, the descending noradrenergic inhibitory tone may be compromised but interestingly, the increase in the α2-AR efficacy can be considered beneficial for the effect of NA reuptake inhibitors.

Although spinal adrenergic alterations have received special attention in the development of chronic pain, supraspinal noradrenergic areas have also been seen to change in a manner that is relevant to chronic pain. Thus, an increase in dopamine beta-hydroxylase (DBH), tyrosine hydroxylase (TH), NAT and α2-ARs has been observed in the LC of rats with long-term neuropathic pain (Alba-Delgado et al., 2013; Tazawa et al., 2015). All these changes might try to balance the reduced spinal noradrenergic tone observed in neuropathic pain, although in recent decades these alterations have been seen to be relevant for the development of secondary co-morbidities in chronic pain, such as anxiety and depression (Alba-Delgado et al., 2013, 2018). It has also recently been demonstrated that neuropathic pain induces LC-BLA overactivation, leading to anxiety-like behaviors, and enhancing the aversive learning and memory index (Llorca-Torralba et al., 2019). In addition, optogenetic activation of the LC-PFC (prefrontal cortex) pathway exacerbates spontaneous pain, producing aversion and increasing anxiety-like behavior (Hirschberg et al., 2017). Interestingly, other noradrenergic nuclei (A5 and A7) contribute to the maintenance of chronic pain. Thus, the A7 seems to exert an inhibitory effect on LC neurons in neuropathic pain (Wei and Pertovaara, 2013), while the A5 nucleus sends projections to the dorsal reticular nucleus (DRt) that contributes to the facilitation of pain transmission in the spinal cord via α1-ARs (Lima and Almeida, 2002; Martins et al., 2015).

### Serotonin

The serotonergic descending pain pathways also experiences alterations in animal models of chronic pain. The effect of spinal 5-HT may be either inhibitory or facilitatory, depending on the acute or chronic state of pain (Millan, 2002). Thus, intrathecal administration of 5-HT produces anti-nociception against acute stimuli of diverse nature (Crisp et al., 1991; Bardin et al., 1997), whereas the depletion of 5-HT pathways in models of neuropathic (chronic) pain prevents hypersensitivity (Suzuki et al., 2004; Rahman et al., 2006). Interestingly, reduced spinal 5-HT levels in mice deficient for the 5-HT transporter (5-HTT^–/–^) is associated with decreased thermal hypersensitivity, a symptom caused by peripheral sensitization in pain (Vogel et al., 2003). Thus, it has been suggested that 5-HT might facilitate persistent pain while it has also been proposed that 5-HT could be inhibitory in neuropathic pain.

Regarding 5-HT receptor stimulation, 5-HT1As have been implicated in both facilitatory (i.e., pronociception; Alhaider and Wilcox, 1993) and inhibitory pain (i.e., anti-nociception; Bardin et al., 2001). The activation of 5-HT1A autoreceptors at the supraspinal levels regulates 5-HT release to the spinal cord and therefore, acute 5-HT1A agonist administration dampens 5-HT release in the spinal cord (Sprouse and Aghajanian, 1987). In animal models of neuropathic pain, the antagonism of 5-HT1A receptors induces analgesia or it potentiates the effect of morphine, tramadol and anti-depressants (Rojas-Corrales et al., 2000, 2005; Ardid et al., 2001; Anjaneyulu and Chopra, 2004), suggesting a facilitatory role for this receptor in neuropathic pain at least. By contrast, chronic administration of the 5-HT1A agonist F-13640 induced analgesia in an animal model of constriction injury of the infraorbital nerve (Colpaert, 2006; Colpaert et al., 2006). This response could be due to the desensitization of autoreceptors in supraspinal areas following chronic 5-HT1A administration. Therefore, the pro-nociceptive effects of 5-HT1A agonist administration have been attributed to pre-synaptic 5-HT1A, while anti-nociceptive effects could be due to the post-synaptic 5-HT1A activation at the spinal level (Colpaert, 2006).

Antagonism of 5-HT2 and 5-HT3 receptors is thought to dampen analgesia or potentiate the effect of some drugs due to its role in descending facilitation pain (Ali et al., 1996; Green et al., 2000). Interestingly, there are increases in the prominence of these receptors in association with chronic pain. Thus, an increase of 5-HT2A receptor mRNA was evident in DRGs, the RMN, ventrolateral PAG (vlPAG), dorsal raphe nucleus (DRN) and dorsal horn in a model of inflammatory pain (Zhang et al., 2001, 2002). Similarly, an increase in the 5-HT2A receptor has been reported in the lumbar dorsal horn in association with neuropathic pain (Thibault et al., 2008). Clinical studies reported an increase in 5-HT2A receptor binding in the PFC related to the pain evoked by heat (Kupers et al., 2009), suggesting a clear facilitation role for this receptor. As such, antagonism of this receptor could be a potential target for the treatment of chronic pain.

Although 5-HT3 receptor expression remains unaltered in the dorsal spinal horn in neuropathic pain (Peters et al., 2010), antagonism of the 5-HT3 receptor reduces the second but not the first phase of the formalin test, suggesting that the agonism of the 5-HT3 receptor would facilitate chronic pain (Glaum et al., 1988, 1990; Green et al., 2000). In addition, an increase in serotonergic fibers (5 weeks after spinal cord injury -SCI) was proposed to contribute to the maintenance of mechanical allodynia via 5-HT3 receptor activation (Oatway et al., 2004). These findings suggest that 5-HT3 and 5-HT2A receptors that participate in facilitatory descending pain contribute to the central sensitization in chronic pain conditions (Bannister and Dickenson, 2017).

There is little information available from a clinical setting, although a decrease in mechanical allodynia was observed in some patients with neuropathic pain treated with a 5-HT3 receptor antagonist (McCleane et al., 2003) but not in others (Tuveson et al., 2011). Although selective 5-HT3 receptor antagonists (used as anti-emetics) have inflammatory and analgesic properties in patients suffering inflammatory rheumatic diseases and fibromyalgia (Stratz and Muller, 2000; Vergne-Salle et al., 2011), there are no clinically effective drugs that act on these receptors.

The 5-HT7 receptor has been little studied in chronic pain. While it is thought to be less prominent in the dorsal spinal cord of neuropathic rats, its antagonism seems to produce analgesic properties in neuropathic and inflammatory pain models (Rocha-González et al., 2005; Amaya-Castellanos et al., 2011; Bannister and Dickenson, 2017).

Thus, it is difficult to elucidate the role of 5-HT and its receptors in pain because such effects are highly dependent on the 5-HT receptor subtype stimulated but also, on the pathological state of the subject.

### Dopamine

Interest in the role of the dopaminergic system in pain has augmented in the last decade. Some changes in the mesocorticolimbic circuit have been reported, considered as a possible target to combat the negative affective states associated with chronic pain. A decrease in D2 receptor expression has been reported in the nucleus accumbens (NAc) of animal models of neuropathic pain, as well as changes in D2 and D3 receptor expression in the hippocampus (Cardoso-Cruz et al., 2014; Sagheddu et al., 2015). In addition, a reorganization of NAc connectivity with cortical areas contralateral (to the injured limb) has been reported in a model of long-term neuropathic pain (Chang et al., 2014). The dopaminergic alterations studied seem to contribute to the negative motivational-affective (Taylor et al., 2003; Scott et al., 2006; Chang et al., 2014) and cognitive aspects of pain (Cardoso-Cruz et al., 2014).

### Histamine

The histaminergic system seems play an important role in the development of hypersensitivity in neuropathic pain (Obara et al., 2019). Histamine is released peripherally by mast cells that are involved in the inflammatory process through the recruitment of macrophages and neutrophils following nerve injury (Calvo and Bennett, 2012). In fact, mast cell depletion prevents mechanical hypersensitivity in a mouse model of persistent pain (Kaur et al., 2017). These mast cells proliferate and can degranulate for up to 2 weeks or even for months under certain conditions of nerve injury (Hayashi et al., 2008). In terms of the histamine receptors, an increase in H1 receptor expression has been observed in nociceptive afferent neurons after nerve injury (Baron et al., 2001; Kashiba et al., 2001) and interestingly, histamine seems to activate the pruriceptors that induce the release of inflammatory mediators involved in pruritus (itching: Parsons and Ganellin, 2006). There is also evidence indicating that histamine-induced itching can convert into pain associated with neuropathic hypersensitivity (Baron et al., 2001). Thus, the histaminergic system may be subject to alterations that contribute to the maintenance of chronic pain.

## Analgesic Drugs With Monoaminergic Action

### Antidepressants

The analgesic activity of antidepressants has been widely demonstrated in animals and humans. Although antidepressants can be used to alleviate certain types of chronic pain, mainly neuropathies, their mechanism of action as analgesics remain unclear (Obata, 2017). Given the central role of monoamines in pain modulation it is highly likely that antidepressants exert some effect on pain circuits. The hypotheses regarding the analgesic activity of these drugs focuses on their ability to augment the presence of monoamines by inhibiting NA and 5-HT reuptake. Antidepressants block NA and 5-HT transporters, preventing presynaptic reuptake and thereby increasing the post-synaptic NA and/or 5-HT that reinforces descending pain inhibitory pathways (Hache et al., 2011; Obata, 2017). However, other actions on the monoaminergic system have also been described for these drugs, as described below (Table 2).

**TABLE 2:** Antidepressants with analgesic action via monoaminergic system.

	**Drugs**	**Effective Dose**	**Model of pain**	**Nociceptive test**	**Effect**	**Receptors**	**Effect antagonized by**	**Animal**	**References**
**Noradrenaline**									
Acute pain	Amitriptyline	15 mg/Kg i.p.		Hot plate	↑ Latency	α2-AR	Reserpine (2 mg/kg i.p.)	Mice	Ghelardini et al., 2000
	Imipramine		Abdominal constriction	Acetic acid test	↓ No constriction		Yohimbine (3 mg/kg i.p.)		
						α2A-AR	BRL 44408 (1 mg/kg i.p.)		
	(+) Oxaprotiline	10, 35 mg/kg i.p.	Abdominal constriction	Acetic acid test	↓ No constriction	α2-AR	RX821002 (1 mg/kg s.c.)	Mice	Gray et al., 1999
	Paroxetine	10 mg/kg i.p.							
	Sibutramine	5, 25 mg/kg i.p.							
	Dothiepine	5, 30 mg/kg i.p.							
	Amitriptyline	5, 25 mg/kg i.p.							
	TCA: Amitriptyline	10 mg/kg i.p.	Heat stimulation	Tail-flick test	Not displays analgesic effect	α2-AR		α2-AR KO Mice	Ozdogan et al., 2004
	Desipramine	4, 20, 40 mg/kg i.p.		Hot plate test	↑ Latency	β1-AR	CGP20712A (1 mg/kg s.c.)	Mice	Mico et al., 1997
	Nortriptyline	2, 4, 20, 40 mg/kg i.p.	Heat stimulation	Tail flick test	↑ Latency	β2-AR	ICI118551 (30 μg/kg s.c.)		
			Abdominal constriction	Acetic acid test	↓ No constriction				
			Inflammatory	Formalin test (first phase)	↓ licking time				
Chronic pain	Venlafaxine	40 mg/kg i.p.	Neuropathic: CCI	Plantar test	↑ Latency	α2-AR	Yohimbine (5 mg/kg i.p.)	Rat	Hajhashemi et al., 2014
		10, 20 mg/kg i.p. (14 days)		von Frey test	↑ Withdrawal threshold				
	SNRI: Duloxetine	10 mg/kg s.c. (3 days)	Neuropathic: SNL	von Frey test	↑ Withdrawal threshold	α2-AR	Idazoxan (30 μg/20 μg i.t.)	Rat	Ito et al., 2018
	Nortriptyline, Imipramine	5 mg/kg i.p.	Inflammatory	Formalin test (late phase)	↓ Licking time	α1-AR	Prazosin (1 mg/kg i.p.; 5 μg, i.c.v.)	Rat	Yokogawa et al., 2002
	Nisoxetine	2.5 mg/kg i.p.							
	Maprotiline	10 mg/kg i.p.							
	Milnacipran	5 mg/kg i.p.							
	Fluvoxamine	20 mg/kg i.p.							
	Mianserin	30, 45 mg/kg o.p. (14 days)	Neuropathic: STZ	Randall-Selitto	↑ Withdrawal threshold	α2-AR	Phentolamine (5 mg/kg o.p.; 14 days)	Rat	Ücel et al., 2015
				Von Frey	↑ Withdrawal threshold	β-AR	Propranolol (5 mg/kg o.p. 14 days)		
				Plantar test	↑ Latency				
				Hot plate test	↑ Latency				
	Nortriptyline	5 mg/kg i.p. (2–3 weeks)	Neuropathic: STZ	von Frey test	↑ Withdrawal threshold	β2-AR	ICI118551 (2 mg/kg i.p.)	Mice	Choucair-Jaafar et al., 2009
	Desipramine	5 mg/kg i.p. (4 weeks)	Neuropathic: SNC	von Frey test	↑ Withdrawal threshold	β2-AR	ICI118551 (2 mg/kg i.p.; 3 days)	β2-AR +/+ Mice	Yalcin et al., 2009
	Venlafaxine	10 mg/kg i.p. (4 weeks)						β2-AR −/− KO Mice	
	Reboxetine	0.8 mg/kg i.p. (4 weeks)							
	Nortriptyline	5 mg/kg i.p. (2–3 weeks)	Neuropathic: SNC	von Frey test	Not displays analgesic effect	β2-AR	Terbutaline (0.5 mg/kg i.p.)	β2AR −/− Mice	Bohren et al., 2013
	Venlafaxine				↑ Withdrawal threshold			C57BL/6J Mice	
**Serotonin**									
Acute pain	Venlafaxine	80 mg/kg i.p.		Hot plate test	↑ Latency	5-HT1A	8-OH-DPAT (0.062 mg/kg s.c.)	Mice	Berrocoso and Mico, 2007
	Fluoxetine	10 mg/kg i.p. (7 days)	Abdominal constriction	Acetic acid test	↓ No constriction	5-HT1A	Pindolol (10 mg/kg i.p.)	Mice	Singh et al., 2001
	Fluoxetine	0.3–1 nmol/paw	Inflammatory	Formalin test (first phase)	↑ Fliching			Rat	Cervantes-Durán et al., 2013
	Paroxetine	5, 10, 20 mg/kg i.p.	Abdominal constriction	Acetic acid test	↓ No constriction	5-HT3	Ondansetron (0.1 mg/kg)	Mice	Kesim et al., 2005
	Fluvoxamine	30 mg/kg i.p.		Paw pressure test	↑ Latency	5-HT3	Granisetron (1 mg/kg, s.c)	Mice	Honda et al., 2006
Chronic pain	Fluvoxamine	10, 30 mg/kg i.p; 100 mg i.t.	Neuropathic: Partial sciatic nerve injury	von Frey test	↑ Withdrawal threshold	5-HT2A/AC	Ketanserin (3 mg/kg i.p.; 10 mg i.t.)	Mice	Honda et al., 2006
	Fluoxetine	0.3–1 nmol/paw		Formalin test (late phase)	↑ Fliching	5-HT2A	Ketanserin (10 pmol/paw)	Rat	Cervantes-Durán et al., 2013
						5-HT2B	RS-127445 (10 pmol/paw)		
						5-HT2C	RS-102221 (10 pmol/paw)		
						5-HT3	Ondansetron (10 nmol/paw)		
						5-HT4	GR-113808 (100 fmol/paw)		
						5-HT6	SB-258585 (10 pmol/paw)		
						5-HT7	SB-269970 (1 nmol/paw)		
		3 nmol/paw and i.t.			↓ Fliching	5-HT1A	WAY-100635 (1 nmol i.t.)		
						5-HT1B/1D	GR-127935 (1 nmol i.t.)		
						HT1B	SB-224289 (1 nmol i.t.)		
						5-HT1D	BRL-15572 (1 nmol i.t.)		
	Imipramine	10 mg/kg i.p.	Inflammatory	Formalin test (late phase)	↓ Licking time	5-HT3	Ondansetron (1 mg/kg i.p.)	Rat	Yokogawa et al., 2002
	Nortriptyline	5, 10 mg/kg i.p.				5-HT2	Ketanserin (1, 2 mg/kg i.p; 60 μg i.c.v.)		
	Nisoxetine	2.5 mg/kg i.p.							
	Maprotiline	10 mg/kg i.p.							
	Milnacipran	5 mg/kg i.p.							
	Fluvoxamine	20 mg/kg i.p.							
	Imipramine	2.5 mg/kg i.p.		Formalin test (late phase)	↑ Licking time	5-HT4	SDZ-205, 557 (0.1 mg/kg i.p.)		
	Fluoxetine	10 mg/kg i.p. (3 weeks)	Neuropathic: STZ	Randall-Selitto	↑ The vocalization thresholds	5-HT2A	TAT-2ASCV peptide (30 ng/rat i.t.)	Rat	Pichon et al., 2010
				Hot plate	↑ Latency				
	Fluoxetine	10, 20 mg/kg i.p.	Neuropathic: STZ	Tail inmersion	↑ Latency	5-HT2A/2C	Ritanserin (1, 2 mg/kg i.p.)	Mice	Anjaneyulu and Chopra, 2004
				Hot-plate test	↑ Latency				
	Clomipramine	6 mg/kg i.v.	Neuropathic: CCI, STZ	Randall-Selitto	↑ Vocalization thresholds	5-HT1A	WAY 100, 635 (0.5, 8 mg/kg s.c.)	Rat	Ardid et al., 2001
	Venlafaxine	5, 10, 20 mg/kg i.v.	Neuropathic: CCI	Randall-Selitto	↑ Vocalization thresholds	5-HT1A	OH-DPAT (0.5 mg/kg s.c.)	Rat	Hernandez et al., 2004
							Antisense oligodeoxynucleotide (0.1 nmol/μl)		
**Dopamine**									
Acute pain	Nomifensine	1.25, 2.5, 5, 10 mg/kg s.c		Tail inmersion	Not displays analgesic effect	D2	Eticlopride (181.3–270 mg/kg ip)	Rat	Gilbert and Franklin, 2001
				Hot plate	Not displays analgesic effect				
				Formalin test	↓ Licking time				
Chronic pain	Amitriptyline	3–30 mg/kg i.p.	Neuropathic: SNL	von Frey test	↑ Withdrawal threshold	D2	Sulpiride (30 μg i.t.)	Rat	Chen et al., 2017
	Milnacipran								
	Duloxetine								
	Fluoxetine								

*AR, adrenoceptor; CCI, chronic constriction injury; i.p., intraperitoneal injection; i.v, intravenous injection; i.t. intrathecal injection; s.c., subcutaneous injection; SNC: sciatic nerve cuffing; SNL, spinal nerve ligation; STZ, streptozotocin-induced diabetic; o.p., oral administration.*

### Action on the Noradrenergic System

#### Preclinical Studies

Animal studies suggest that α2-ARs are involved in the analgesia mediated by antidepressants (Ghelardini et al., 2000; Hajhashemi et al., 2014), the most relevant evidence provided by the loss of analgesia induced by amitriptyline in α2-AR KO mice (Ozdogan et al., 2004). In addition to α2-ARs, other adrenergic receptors have also been implicated in the analgesia mediated by antidepressants. Thus, the α1-AR antagonist prazosin blocked the anti-nociceptive effect of nortriptyline, maprotiline, milnacipran, and imipramine in the formalin test (Yokogawa et al., 2002). In terms of β-ARs, initial studies reported that β1- and β2-ARs participated in the analgesia mediated by desipramine (DMI) and nortriptyline, in both thermal and chemical models of acute pain (Mico et al., 1997). Interestingly, in neuropathic mice lacking β2-ARs chronic treatment with DMI, nortriptyline and reboxetine failed to produce analgesia (Yalcin et al., 2009), consistent with preclinical findings where repeated stimulation of β2-ARs seem to be necessary and sufficient to produce a therapeutic effect (Choucair-Jaafar et al., 2009, 2011; Yalcin et al., 2009, 2010). More recently, mianserin, a tetracyclic antidepressant, was shown to produce analgesia in a model of diabetic neuropathic pain through its interactions with both α2- and β-ARs (Ücel et al., 2015). In addition, antidepressants may possibly have peripheral effects, with nortriptyline and venlafaxine mediating the stimulation of β2-ARs in non-neuronal cells, thereby inhibiting the production of the cytokine tumor necrosis factor α (TNFα), provoking an anti-allodynic effect in a rat model of neuropathic pain (Bohren et al., 2013).

All the data available suggest that the analgesic action of antidepressants mainly occurs at the spinal level (Mico et al., 2006; Ito et al., 2018; Kremer et al., 2018). Indeed, it was recently verified that daily subcutaneous injections of duloxetine attenuate SNL hypersensitivity in rats through NA accumulation in the spinal cord (Ito et al., 2018). Given the role of the LC in analgesia through the descending pain pathway, the effect of antidepressants at this level has been evaluated. As expected, electrophysiological assays have demonstrated the action of antidepressants on the LC neurons, for example the acute administration of venlafaxine (a SNRI) completely inhibits the LC activity modulated by α2-ARs and 5-HT1A receptors (Berrocoso and Mico, 2007). Interestingly, DMI and duloxetine restored the functional changes in rat LC neurons affected by neuropathic pain, an event that correlated with analgesic behavior (Alba-Delgado et al., 2012). Similarly, functional Magnetic Resonance Imaging (fMRI) showed that chronic administration of DMI (14 days, daily i.p. of 10 mg/kg) produced greater activation of areas altered by chronic pain in a rat model of neuropathic pain: primary somatosensory cortex, insular cortex, medial globus pallidus, etc. (Jones et al., 2009).

#### Clinical Approach

Antidepressants that mainly inhibit noradrenaline reuptake, like DMI and maprotiline, are not a good choice for the treatment of human neuropathic pain (Max et al., 1991, 1992; Vrethem et al., 1997), yet they are used in patients who have not obtained pain relief with other treatments. Clinical evidence suggests that dual antidepressants provide better analgesic efficacy in chronic neuropathic pain. In this regard, the analgesic efficacy of amitriptyline (a tricyclic antidepressant -TCA) is the best-documented in complex regional pain syndrome, neuropathic and musculoskeletal pain, and fibromyalgia (Wiffen et al., 2013; Moore et al., 2015; Brown et al., 2016; van den Driest et al., 2017). However, due to the side effects of TCAs, the SNRI duloxetine has been the first choice treatment for peripheral diabetic neuropathic pain and fibromyalgia, with a good safety profile. Despite the efficacy revealed in these conditions, this class of antidepressants is not useful in relieving SCI, phantom limb and HIV neuropathy (Kieburtz et al., 1998; Cardenas et al., 2002; Robinson et al., 2004), yet there is insufficient evidence to prescribe antidepressants for inflammatory chronic pain (Perrot et al., 2006; Richards et al., 2011). Thus, more clinical trials are needed in this area.

Regarding the mechanisms of action, it is believed that the increase of monoamines associated with the inhibition of NA and 5-HT reuptake reinforces inhibitory descending pain pathways (Obata, 2017). Clinical studies have reported the importance of stimulating α2A-AR to induce analgesia and indeed, α2 agonist administration has anti-nociceptive activity alone or in combination with opioids (Eisenach et al., 1994, 1995). In clinical practice, antidepressants exhibit little or only intermediate efficacy against acute nociceptive stimuli, while they induce significant analgesia in chronic pain (Poree et al., 1998; Sawynok et al., 2001; Paqueron et al., 2003; Mico et al., 2006). Interestingly, enhanced α2A-AR efficiency could explain why antidepressants induce spinal analgesia in chronic pain (Luo et al., 1994; Malmberg et al., 2001; Bantel et al., 2005). Accordingly, drugs acting through α2-ARs such as clonidine is effective in patients with neuropathic pain at doses that are inactive in post-operative pain (Eisenach et al., 1995). Although preclinical evidence indicates that β2-ARs are necessary to produce a therapeutic effect (Choucair-Jaafar et al., 2009, 2011; Yalcin et al., 2009, 2010), there is one clinical report supporting the efficacy of β2-ARs in combating neuropathic pain (Cok et al., 2010).

### Action on the Serotonergic System

#### Preclinical Studies

Like noradrenaline, the main putative analgesic mechanism for 5-HT is the increase of 5-HT neurotransmission by 5-HT receptor stimulation and the ensuing modulation of descending pain inhibition. However, specific 5-HT receptor subtypes can dampen or enhance neuronal spinal cord activity (Millan, 2002), thereby weakening or potentiating descending pain transmission. Therefore, the role of 5-HT in analgesia remains controversial.

The effect of selectively inhibiting 5-HT reuptake has mainly been studied using SSRIs like fluoxetine. The anti-nociceptive effect of fluoxetine has been demonstrated in several animal models of acute and chronic pain (tail flick, hot plate and streptozotocin diabetic mice), although the data obtained have at times been contradictory (Singh et al., 2001; Cervantes-Durán et al., 2013). Thus, 5-HT has a pro-nociceptive peripheral effect, as reflected by the enhanced pain in the formalin test after local fluoxetine injection. However, systemic and intrathecal injection reduces any pro-nociceptive behavior in this test (Cervantes-Durán et al., 2013). The peripheral pro-nociceptive effect seems to be mediated by the activation of 5-HT2A/2B/2C/3/4/6/7 receptors, while systemic and intrathecal anti-nociception seems to be mediated by 5-HT1A/1B/1D/5A receptor activation.

The pro-nociceptive effect of 5-HT on some 5-HT receptors reveals the importance of co-administering certain 5-HT antagonists in pain management. As such, analgesia is enhanced when 5-HT1A receptors are blocked with drugs that augment 5-HT. Accordingly, the anti-nociceptive effect of the SNRI venlafaxine (injected subcutaneously) was potentiated in the hot plate test by 5-HT1A receptors antagonism. Similarly, systemic co-administration of a 5-HT1A receptor antagonist enhanced fluoxetine-induced anti-nociception in the acetic acid test (Singh et al., 2001). Moreover, several studies showed that systemic blockage of 5-HT1A receptors enhances the analgesic effect of SSRIs like fluoxetine (Anjaneyulu and Chopra, 2004) and clomipramine (Ardid et al., 2001) in chronic pain models. In fact, chronic intracerebroventricular (ICV) administration of an antisense oligodeoxynucleotide that silences 5-HT1A receptor synthesis decreases mechanical hypersensitivity of neuropathic rats (Hernandez et al., 2004). These results provide strong evidence of the importance of co-administering selective 5-HT1A antagonists to potentiate the anti-nociceptive efficacy of antidepressants.

The antagonism of 5-HT2 receptors potentiates the anti-nociceptive effect of paroxetine, effect that is blocked by 5-HT3 antagonist in the acetic acid test (Kesim et al., 2005). However, ketanserin (a 5-HT2A/AC receptor antagonist) antagonizes the anti-allodynic effect of fluvoxamine in a mice model of neuropathic pain. These findings suggest that SSRIs would have an opposite effect on 5-HT2A/2C receptors in acute or chronic pain (Honda et al., 2006).

There is little information about the role of 5-HT receptors in chronic pain and despite the influence of 5-HT on pain modulation, some preclinical studies have reported that the analgesia produced by SSRIs is less potent in chronic than in acute pain models (Bardin et al., 2000; Pichon et al., 2010). In fact, the stimulation of 5-HT2A receptors induces analgesia in healthy but not diabetic neuropathic rats. Thus, the alterations to 5-HT2A receptors that occurs in neuropathic pain is thought to be one of the causes of this inefficacy. In fact, pharmacological disruption of the association between spinal serotonin type 2A (5-HT2A) receptors and their associated PDZ proteins suppresses the analgesia induced by fluoxetine in diabetic and SNL rats (Pichon et al., 2010). Hence, 5-HT2A receptors and PDZ protein interactions might influence the resistance to SSRI-induced analgesia in the management of chronic neuropathic pain. Coupling the pro-nociceptive stimulation of some 5-HT receptors to the 5-HT alterations observed in chronic pain possibly explains the inefficacy of SSRIs in the management of chronic pain.

#### Clinical Approach

Although modulation of the 5-HT system could dampen or enhance the magnitude of pain, antidepressants like SSRIs that selectively acting through 5-HT are thought to be relatively poor in combating chronic pain (Johansson and Von Knorring, 1979; Anderberg et al., 2000) and therefore, they are not recommended as a first-line therapy for chronic pain. Fluoxetine and paroxetine have been used unsuccessfully to manage non-neuropathic chronic pain like headache and migraine (Saper et al., 1994, 1995), and paroxetine and citalopram have minimal effects on patients suffering painful diabetic neuropathy. Similarly, fluoxetine had no effect in patients with painful diabetic neuropathy (Sindrup et al., 1990, 1992; Max et al., 1992). Animal studies have helped us to elucidate some possible mechanisms responsible for the ineffectiveness of SSRIs in chronic pain, such as the facilitatory role of serotonin on 5-HT3 receptors and the alterations to 5-HT2A receptors in this condition. However, other drugs with dual action on NA and 5-HT reuptake inhibitors have proved to be efficacious in treating several forms of chronic pain, such as venlafaxine and duloxetine for fibromyalgia, migraine and diabetic neuropathy (Kaur et al., 2011; King et al., 2015; Burch, 2019). Hence, further studies should be carried out to assess how to achieve effective analgesia through the 5-HT system. By contrast, there is no evidence to support the use of SSRIs to combat inflammatory chronic pain like rheumatoid arthritis or osteoarthritis (Perrot et al., 2008).

### Action on the Dopaminergic System

#### Preclinical Studies

The descending release of DA in the spinal cord plays an important role in pain modulation, although there are limited studies into the dopaminergic influence on the analgesic effect of antidepressants. Intrathecal injection of bupropion produces a dose-dependent anti-hyperalgesic effect in neuropathic rats, coincident with an increase in NA and DA in the spinal cord (Hoshino et al., 2015). In addition, the norepinephrine-dopamine reuptake inhibitor nomifensine has a similar potency and efficacy to morphine in the formalin test (Gilbert and Franklin, 2001). It was recently reported that spinal DA receptors are implicated in the anti-hyperalgesic effect of antidepressants in a model of spinal nerve ligation. Pretreatment with a dopamine D2 receptor antagonist abolished the anti-hyperalgesic effects of intraperitoneal amitriptyline, duloxetine, milnacipran and fluoxetine administration (Chen et al., 2017), suggesting that DA would exert a role in the spinal inhibitory effect of antidepressants. Nevertheless, further studies into the implication of the DA system in pain should be carried out.

#### Clinical Approach

Few studies have evaluated the analgesic effectiveness of specific DA reuptake inhibition in chronic pain patients. In this regard, the atypical antidepressant bupropion (a dual noradrenaline and dopamine reuptake inhibitor) decreases the intensity of pain and improves the quality of life of neuropathic pain patients (Semenchuk and Davis, 2000). However, the side effects of this drug are an important drawback as bupropion may be an excessively strong stimulant, inhibiting the appetite. As such, it is absolutely contraindicated in patients with a history of seizures or eating disorders.

Nonetheless, new TRIs have been designed that block all three monoamines by acting on their transporters (DAT, NAT and SERT: dopamine, noradrenaline, and serotonin transporters: Hache et al., 2011). These drugs should theoretically be very efficacious in treating chronic pain and they could be useful when pain is co-morbid with other conditions in which the availability of some monoamines is compromised, such as in depression or Parkinson’s disease. However, whether these drugs truly represent an interesting new strategy remains to be fully explored.

### Gabapentinoids

#### Preclinical Studies

Gabapentin was introduced as an antiepileptic drug in 1993 and it was subsequently recognized as a first-line drug for several types of chronic pain. Gabapentinoids have been widely used to treat neuropathic pain and they are mainly characterized by their action on α2δ subunits in primary afferents. However, they may also act on supraspinal areas and stimulate descending pain inhibition, with their supraspinal effects mediated by spinal α2-ARs (Table 3; Takasu et al., 2006). Accordingly, high doses of gabapentin and pregabalin (ICV), and i.v. administration of gabapentin, enhances NA turnover in the spinal cord (Takeuchi et al., 2007; Hayashida et al., 2008; Tanabe et al., 2008), provoking spinal α2-AR mediated analgesia. In murine models of neuropathic pain, α2 antagonists suppress the anti-hypersensitivity effect of gabapentinoids following ICV administration (Tanabe et al., 2008). There have been few studies into gabapentinoids and the serotonergic system, yet the increase in 5-HT3 receptors after injury determines the analgesic actions of gabapentinoids (Bee and Dickenson, 2008). Conversely, pregabalin induced pain relief via the intra-accumbens nucleus induces DA release during early but not late neuropathic pain (Kato et al., 2016). Hence, pain chronification leads to dysfunction of the reward circuit, which should be studied as a target for the management of chronic pain.

**TABLE 3:** Analgesic action of gabapentinoids, opioids, NSAID’s and histaminergic drugs via monoaminergic system.

**Drugs**	**Effective Dose**	**Model of pain**	**Nociceptive test**	**Effect**	**Receptors**	**Effect antagonized by**	**Animal**	**References**
**Gabapentinoids**								
Gabapentin	100 μg i.c.v.	Neuropathic: PSL	Plantar test	↑ Latency	α2-AR	Yohimbine (3 μg, i.t.)	Mice	Takasu et al., 2006
			von Frey test	↑ Latency		Idazoxan (3 μg, i.t.)		
Gabapentin	1 μg intra LC	Neuropathic: SNL	Randall-Selitto	↑ Latency	α2-AR	Idazoxan (30 μg, i.t.)	Rat	Hayashida et al., 2008
Gabapentin	100 μg i.c.v.	Neuropathic: PSL	Plantar test	↑ Latency	α2-AR	Idazoxan (3 μg, i.t.)	Mice	Takeuchi et al., 2007
Pregabalin	30 i.c.v.		von Frey test	↑ Latency	α2-AR			
Gabapentin	100 mg/kg i.p.	Neuropathic: PSL	Plantar test	↑ Latency	α2-AR	Yohimbine (1 mg/kg i.p.; 3 μg i.t)	Mice	Tanabe et al., 2008
	100 μg i.t.		von Frey test	↑ Latency				
**Opioid**s								
Beta-endorphin	1 nmol / 10 μl i.t.	Heat stimulation	Tail-flick test	↑ Latency	5-HT1	Spiroxatrine (15 μg/10 μl)	Rat	Crisp et al., 1989
					5-HT3	ICS 205-930 (15 μg/10 μl)		
					5-HT2	Ritanserin (15 μg/10 μl)		
					α2-AR	Yohimbine (15 μg/10 μl)		
DAMGO	1 μg/paw	PGE2 (intraplantar)	Randall-Selitto	↑ Nociceptive threshold	α2-AR	Yohimbine (5, 10, 20 mg/paw)	Rat	Romero et al., 2012
SCN 80	20 μg/paw				α2A-AR	BRL 44480 (20 μg/paw)		
Bremazocine	20 μg/paw				α2B-AR	Imiloxan (20 μg/paw)		
					α2C-AR	RAU (10, 15, and 20 μg/paw)		
					α2D-AR	RX 821002 (20 μg/paw)		
					α1-AR	Prazosin (0.5, 1, 2 μg/paw)		
					β-AR	Propanolol (150, 300, 600 ng/paw)		
Morphine	7.5 nmol/10 μl i.t	Heat stimulation	Tail-flick test	↑ Latency	5-HT2	Ketanserin (50 nmol, i.t.)	Rat	Kellstein et al., 1988
						Methysergide (20 nmol i.t.)		
Fluoxetine	10 mg/kg i.p. (7 days)	Abdominal constriction	Acetic acid test	↓ Writhes	5-HT1A	Naloxone (5 mg/kg i.p.)	Mice	Singh et al., 2001
						Naltrexone (5 mg/kg i.p.)		
**NSAID’s**								
Paracetamol	400 mg/kg i.p.		Hot plate test	↑ Reaction time	5-HT2	[3H]Ketanserin binding	Rat	Pini et al., 1996
ASA+morphine	50 mg/kg i.p; 3 mg/kg s.c	Inflammatory	Formalin test (both phases)	↓ Flinches				
Clonidine	1, 10 μg i.t.	Heat stimulation	Noxious heat stimuli	↑ Latency	α2-AR	Ketorolac (50 μg i.t.)	Rat	Conklin and Eisenach, 2003
Dypirone	20 μg/paw	PGE2 (intraplantar)	Randall-Selitto	↑ Latency	α2-AR	Yohimbine (2.5, 5, 10, 20 mg/paw)	Rat	Silva et al., 2015
Diclofenac	40 μg/paw				α2C-AR	RAU (10, 15, 20 μg/paw)		
					α1-AR	Prazosin (0.5, 1, 2 μg/paw)		
					β-AR	Propanolol (0.3, 0.6, 1.2 μg/paw)		
**Histamines**								
Chlorpheniramine	15 mg/kg i.p.	Neuropathic: TNT	von Frey test	↑ Latency	H1		Rat	Khalilzadeh et al., 2018
Ranitidine	15 mg/kg i.p.		Acetone test	↑ Latency	H2			
Histidine	100 mg/kg, i.p.	Neuropathic: PSL	von Frey test	↑ Latency	H1	Mepyramine (200 ng/rat i.t. or i.c.v)	Rat	Yu et al., 2016
			Plantar test	↑ Latency				
Promethazine	50, 100 mg/kg, i.p. (12 days)	Vincristine-mediated neuropathy	Pinprick test	↑ Latency	H1		Rat	Jaggi et al., 2017
Ranitidine	20, 20 mg/kg, i.p. (12 days)		Acetone test	↑ Latency	H2			
			Hot plate test	↑ Latency				
E-162	1, 5, 10, 20 mg/kg, i.p.	Neuropathic: CCI	von Frey test	↑ Latency	H3	Pyrilamine (H1 receptor antagonist, 10 μg i.t.)	Mice	Popiolek-Barczyk et al., 2018
TR-7			Cold plate	↑ Latency	H4			
			Tail-flick test	↑ Latency				
S38093	1 mg/kg, p.o. (4 days)	Neuropathic: CCI	Randall-Selitto	↑ Latency	H3		Rat	Chaumette et al., 2018
A-960656	1, 3 mg/kg, p.o. (11 days)	Neuropathic: SNL	Randall-Selitto	↑ Latency	H3		Rat	Cowart et al., 2012
	0.1, 0.3, 1 mg/kg, p.o. (12 days)	Inflammatory						
JNJ7777120	28, 70 mg/kg i.p. (8 days)	Neuropathic: SNL	Plantar test	↑ Latency	H4		Rat	Hsieh et al., 2010
		Inflammatory						
ST-1006	30 and 60 μg i.c.v.	Neuropathic: SNI	Plantar test	↑ Latency	H4	JNJ 10191584 (6 mg/kg p.o.)	Mice	Sanna et al., 2015, 2017
VUF8430	20 and 40 μg i.c.v.		Von Frey test	↑ Latency				

*AR, adrenoceptor; ASA, Acetylsalicylic acid; CCI, chronic constriction injury; i.c.v, intracerebroventricular injection; i.p., intraperitoneal injection; i.t. intrathecal injection; p.o, oral administration; PSL, partial ligation of the sciatic nerve; s.c., subcutaneous injection; SNL, spinal nerve ligation; TNT, tibial nerve transection; SNI, spared nerve injury.*

#### Clinical Approach

Gabapentinoids are mainly effective in the treatment of chronic neuropathic pain, with gabapentin and pregabalin the drugs considered to most effectively relieve neuropathic pain (e.g., diabetic neuropathy and post-herpetic neuralgia). However, in terms of non-neuropathic pain only pregabalin has been seen to be efficacious in the treatment of fibromyalgia (Wiffen et al., 2013). Most clinical trials indicate that antidepressants and gabapentinoids produce comparable analgesia, mainly in neuropathic pain patients, yet more recent trials attempted to see if analgesia is improved by combining these two drugs. For neuropathic pain (diabetic neuropathy or post-herpetic neuralgia), the combined action of gabapentin and the TCA nortriptyline proved to be more efficacious than either drug alone (Gilron et al., 2009). The combination of pregabalin and duloxetine was well tolerated in diabetic neuropathy patients, although it did not produce better analgesia than either drug alone (Tesfaye et al., 2013). Interestingly, this combination produced a notably better clinical outcome in fibromyalgia when compared to monotherapy (Gilron et al., 2016). There is little data regarding the use of gabapentinoids in pain with a pure inflammatory component, although a recent study indicated that the combination of pregabalin with a NSAID in knee osteoarthritis patients with neuropathic pain was more effective than monotherapy with the NSAID alone (Filatova et al., 2017).

### The Combination of Monoamines and Opioids

#### Preclinical Studies

Opioids are first line treatments for many types of chronic pain and several studies have reported combined effects between the opioid and monoaminergic systems (Table 3; Schroder et al., 2011; Bravo et al., 2017). Opioid receptor agonists induce NA release at supraspinal, spinal and peripheral sites (Wigdor and Wilcox, 1987; Grabow et al., 1999) where they would exert analgesic actions. Early studies reported that α2-ARs mediate analgesia of β-endorphin at the spinal cord (Crisp et al., 1989) and that naloxone antagonizes intrathecal NA-induced anti-nociception, suggesting that opioids are involved in NA spinal analgesia. Interestingly, co-administration of TCAs and morphine enhance analgesia in mice (Kellstein et al., 1984; Hwang and Wilcox, 1987; Gray et al., 1999; Reimann et al., 1999), and α2-ARs are thought to be responsible for this synergistic action (Stone et al., 1999). Recent studies demonstrated that opioids and cannabinoids induce peripheral anti-nociception by releasing NA, which activates the α2-, α1- and β-ARs (Romero et al., 2012).

However, there are is some controversy as to whether opioids mediate spinal anti-nociception via NA or 5-HT. Inhibition of both NA and 5-HT reuptake appears to induce analgesic synergy with morphine in the formalin test, although excess 5-HT may stimulate 5-HT3 receptors and reduces this synergy (Shen et al., 2013). Alternatively, there is evidence that opioids mediate spinal 5-HT anti-nociception (Kellstein et al., 1988; Kishimoto et al., 2001) and moreover, the anti-nociceptive effect of fluoxetine in the acetic acid test is sensitive to blockade by naloxone (Singh et al., 2001), suggesting a clear opioid component in this effect.

#### Clinical Approach

Combining monoamines and opioids enhances analgesia in animal models, although in only one clinical trial were benefits reported using the combination of nortriptyline and morphine (Gilron et al., 2015). Unfortunately, this combination induces the classic adverse effects of opioids (constipation, dry mouth and somnolence) and could compromise patient safety. Hence, drugs are being designed that offer a reasonable safety profile and that combine monoaminergic and opioid actions. This is the case of tramadol, a mu-opioid agonist and SNRI, and tapentadol, a potent mu-opioid agonist and NA reuptake inhibitor. These drugs relieve certain types of chronic pain but they do not appear to have a reliable effect on neuropathies (Silverfield et al., 2002; Duehmke et al., 2017), which has led to them being more commonly recommend as second-line therapy.

### NSAIDs

#### Preclinical Studies

Preclinical and clinical studies demonstrated the efficacy of NSAIDs, mainly in chronic inflammatory pain (Cohen and Harris, 1987; Vo et al., 2009), and they are commonly prescribed for acute and persistent inflammatory pain. The peripheral inhibition of prostaglandins is not the only analgesic mechanism of action, given its central action on the noradrenergic and serotonergic system (Table 3). Acetyl salicylic acid (ASA) induced analgesia is accompanied by an increase in the turnover of 5-HT, NA and DA (Bensemana and Gascon, 1978), and a potent relationship has been described between 5-HT and NSAIDs. Acute administration of phenazone decreases 5-HT binding, whereas chronic treatment provokes an increase in 5-HT binding sites in the pontine and cortical areas, induced by an increase in 5-HT (Sandrini et al., 1998). The anti-nociceptive effect of ASA was impeded by i.p. pre-treatment with a 5-HT neurotoxin in the hot plate test (Pini et al., 1996). Moreover, ASA potentiated the anti-nociceptive effect of morphine in the second phase of the formalin test, accompanied by an increase in extracellular 5-HT and a decrease in 5-HT2 receptors in the cortex (Sandrini et al., 1998). Similar findings were reported with the combination of paracetamol and morphine. More recently, adrenergic receptors have been implicated in the anti-nociceptive effect of NSAIDs. Thus, intrathecal ketorolac enhances the effect of clonidine against a noxious heat stimulus in rats, yet it lacks efficacy when administered alone (Conklin and Eisenach, 2003). Additionally, the peripheral depletion of NA prevented the anti-nociceptive effect of dipyrone and diclofenac in a model of inflammatory pain, while prazosin (an α1 antagonist) and propranolol (a β-adrenergic antagonist) blocked the anti-nociceptive effect of dipyrone and diclofenac (Silva et al., 2015), suggesting that NSAID analgesia is mediated by adrenergic receptors. There are no relevant studies into the analgesic efficacy of NSAIDs in animal models of neuropathic pain (Lashbrook et al., 1999), yet further preclinical studies into both chronic neuropathic and inflammatory pain should elucidate the possible interaction between NSAIDs and monoamines.

#### Clinical Approach

NSAIDs are routinely prescribed for the management of chronic inflammatory pain since their main mechanism of action involves the peripheral inhibition of inflammatory mediators (prostaglandins), provoking weaker peripheral sensitization. However, the oral NSAIDs used have more severe adverse effects when administered chronically (peptic ulcer disease, acute renal failure and stroke/myocardial infarction) and thus, topical rather than oral administration is recommended. In clinical practice, NSAIDs have relevant peripheral activity but recently, there is recent evidence that they may facilitate activation of the descending inhibitory circuitry at the level of the spinal cord and brain (Hodkinson et al., 2015). In terms of neuropathic pain, NSAIDs have no effect and there is only modest evidence of efficacy in patients with chronic low back pain (Enthoven et al., 2017).

### Histaminergic Drugs

Recent, preclinical findings support the use of ligands of histamine receptors as analgesics in neuropathic and inflammatory pain (Table 3; Obara et al., 2019). For example, the systemic administration of H1 and H2 receptor antagonists (like chlorpheniramine and ranitidine, respectively) produces anti-allodynic effects in a model of neuropathic pain in rats (Khalilzadeh et al., 2018). Interestingly, several studies suggest that H1 but not H2 receptors are involved in the development of hypersensitivity following partial ligation of the sciatic nerve and in vincristine-induced models of neuropathic pain (Yu et al., 2016; Jaggi et al., 2017).

Although most studies suggest that the systemic use of histamine H3 receptor antagonists produces inhibitory effects on nociceptive responses in neuropathic pain (Farzin and Nosrati, 2007; Chaumette et al., 2018; Popiolek-Barczyk et al., 2018), there is evidence that activation of H3 receptors has inhibitory effects on pain (Cannon et al., 2003). Interestingly, chronic oral administration of S38093 or A-960656, selective H3 receptor antagonist/inverse agonists, increased the paw withdrawal threshold to mechanical stimuli in neuropathic and inflammatory pain models, showing similar effects to gabapentin and/or pregabalin (Cowart et al., 2012; Chaumette et al., 2018). Moreover, the analgesic effect of S38093 is thought to be partially mediated by a2-AR desensitization in the LC, suggesting that the noradrenergic system is crucial for H3 antagonists to produce antinociception (Chaumette et al., 2018). In fact, bilateral lesions of the LC and spinal cord transection completely inhibited the effects of H3 antagonist, on spontaneous and evoked firing of spinal neurons in neuropathic rats (McGaraughty et al., 2012). Like H3 receptors, H4 receptor agonists and antagonists have different effects on the nociceptive response. Thus, systemic administration of JNJ 7777120, a histamine H4 receptor antagonist, reduced mechanical hypersensitivity in chronic constriction injury (CCI) and SNL neuropathic models (Hsieh et al., 2010). In addition, single doses of the selective H4 receptor antagonist TR-7 elicit a strong analgesic effect (Popiolek-Barczyk et al., 2018). By contrast, local ICV administration of the H4 receptor agonists ST-1006 and VUF8430, reduced nociceptive thresholds in mice with neuropathic pain (Sanna et al., 2015).

Overall, there appears to be a clear effect of histamine in chronic pain that depends on multiple factors, such as the localization of the receptors, or the affinity and selectivity of ligands for histamine receptors (Obara et al., 2019). Thus, a better understanding of the histaminergic system in pain modulation will help us to identify histaminergic targets that could lead to more efficient pharmacological therapy for neuropathic pain.

## Discussion

The monoaminergic system is important in both the healthy state and in pain modulation, the correct balance between the excitatory and inhibitory inputs on descending pain pathways producing analgesia. However, when biological pain shifts to pathological pain, the monoaminergic system suffers alterations that would contribute to the sensitization and maintenance of pain. To search for pharmacological new targets, the monoaminergic mechanisms underlying chronic pain are of great interest. In fact, chronic pain, and explicitly neuropathic pain, is treated with medications that affect monoamines. Antidepressants are drugs that effect monoamines directly, although they were designed to treat depression and they are currently used as first line treatments for neuropathic pain due to their intrinsic analgesic action. In recent decades, antidepressants have been seen to have greater clinical efficacy in neuropathic pain than in inflammatory pain (Finnerup et al., 2005; Sindrup et al., 2005). From an etiological point of view, neuropathic pain is due to lesion or dysfunction in the PNS/CNS that causes monoaminergic dysfunction, yet inflammatory pain is caused by the action of inflammatory mediators on nociceptors in the periphery and subsequently, changes in the excitability of central neurons. The analgesic mechanism of action of antidepressants is thought to involve inhibition of both NA and 5-HT reuptake at spinal and brain levels, and the subsequent modulation of descending pain pathways. Thus, it is plausible that antidepressants exert positive clinical outcomes in pain with a neuropathic component.

In addition, antidepressants exhibit significant analgesia in chronic pain, yet moderate efficacy against acute pain (Poree et al., 1998; Sawynok et al., 2001; Paqueron et al., 2003; Mico et al., 2006). As indicated, the increase of α2-AR efficacy at the spinal level seems to promote better analgesia of antidepressants (Stone et al., 1999; Bantel et al., 2005). Moreover, supraspinal noradrenergic centers like the LC are altered in chronic pain, alterations that are relevant for the development of chronic pain-related co-morbidities like anxiety and depression (Alba-Delgado et al., 2013, 2018), and which are commonly treated with antidepressants. Although antidepressants mainly act through the NA and 5-HT system, the repercussion of each system on anti-nociception remains unclear. Antidepressants that affect both noradrenaline and serotonin levels, like duloxetine and amitriptyline, are more potent and efficient than SSRIs in relieving chronic pain (Marchand et al., 2003; Finnerup et al., 2005). Considering data from animal studies, milnacipran (a SNRI) but not paroxetine (a SSRI) attenuates mechanical hypersensitivity in a model of neuropathic pain (SNL: Bomholt et al., 2005). Furthermore, the anti-nociceptive effect of drugs that act selectively on 5-HT (e.g., SSRIs) is less potent in chronic than in acute pain (Bardin et al., 2000; Pichon et al., 2010). This phenomenon could be explained by the pro-nociceptive effect of spinal 5-HT receptors in some states of chronic pain and by the alterations to 5-HT2A receptors observed in neuropathic pain (Honda et al., 2006; Pichon et al., 2010). These studies reveal the importance of studying 5-HT receptor agonists/antagonists in pain management. Currently, no drugs used to manage chronic pain target 5-HT receptors, except the 5-HT1B/1D agonists used to treat chronic migraine and headache. Interestingly, drugs that mainly inhibit noradrenaline reuptake, like DMI and maprotiline, are not a good choice for pain relief, as reflected by their moderate relief in the treatment of human neuropathic pain (Max et al., 1991, 1992; Vrethem et al., 1997), revealing the importance of increasing both extracellular NA and 5-HT.

Clinical evidence suggests TCAs are more efficacious in several chronic pain conditions. In particular, amytriptiline is considered the gold standard to manage neuropathic pain irrespective of its etiology (Saarto and Wiffen, 2007), although the main drawback of these drugs are their adverse effects. However, optimum analgesia is usually achieved at lower doses than those required for their antidepressant activity and thus, such side effects occur less frequently. Non-tricyclic antidepressants are thought to be safer than TCAs, and duloxetine is the first antidepressant approved by the FDA to treat neuropathic pain, considered the best election for peripheral diabetic neuropathic pain (Kaur et al., 2011; King et al., 2015). Less convincing results have been obtained in terms of the selective action on 5-HT receptors, yet preclinical studies indicate that the co-administration of antidepressants with certain 5-HT receptor antagonists (e.g., those of the 5-HT1A receptor) potentiates the analgesic effect of these drugs (Ardid et al., 2001; Singh et al., 2001; Anjaneyulu and Chopra, 2004). Recently, other atypical antidepressants like melatonin and agomelatonine have been shown to have promising analgesic effects to treat neuropathic pain, acting via MT1/MT2 melatonin receptors and the monoaminergic system (Ambriz-Tututi et al., 2009; Lopez-Canul et al., 2015; Chenaf et al., 2017). Moreover, the introduction of TRIs represents a new strategy that can be explored in the management of chronic pain. As indicated, chronic pain induces important changes in the NA, 5-HT and DA system, and as such, these drugs would maintain optimal levels of these neurotransmitters that allow a longer interaction with the appropriate receptors.

In addition to antidepressants, other drugs that directly and indirectly effect the monoaminergic system are also used to treat chronic pain. Thus, atypical opioids that combine monoaminergic and opioid effects are more efficacious than other opioids in neuropathic pain models (Christoph et al., 2007; Meske et al., 2014). In fact, tramadol tapentadol are situated on the second third step of the WHO analgesic scale (WHO, Bravo et al., 2017). Conversely, gabapentinoids mediate analgesia via spinal α2-ARs (Takasu et al., 2006) and they have been approved by the FDA to treat neuropathic pain (Goodman and Brett, 2019). The efficacy of gabapentinoids is comparable to that of antidepressant drugs in clinical trials, yet they differ in safety and tolerability (Morello et al., 1999; Sindrup and Jensen, 2000). They now represent an alternative for neuropathic pain patients for whom amytriptiline is contraindicated. Finally, NSAIDs are drugs that act on the monoaminergic system, yet with limited information regarding their mechanism of action. In general, NSAIDs seem to potentiate the analgesic effect of drugs like morphine and tramadol, and recently, their activity was associated with α1- and β-ARs (Silva et al., 2015). Clinically, there is a little evidence of the potency and efficacy of NSAIDs in chronic pain, or of their use in combination with other drugs (Ong et al., 2007). Finally, although there are no histaminergic drugs that relieve chronic pain, preclinical studies indicate that it is time to reconsider the histamine system as a therapeutic target for the management of inflammatory and neuropathic pain.

In conclusion, an extracellular increase in NA and 5-HT significantly helps relieve chronic pain. Indeed, chronic neuropathic pain is commonly treated with TCA and SNRI antidepressants, and by gabapentinoid drugs when antidepressants are contraindicated. Given that the monoaminergic system is closely linked to disorders like depression and anxiety, clinical situations frequently associated with chronic pain, the monoaminergic system is a pharmacological target that could help treat the sensory and emotional aspects of chronic pain. However, despite the promising preclinical and clinical results obtained, complete relief from chronic pain via the monoaminergic system remains a challenge. Thus, there is a need for further preclinical and clinical studies to further assess the selective targeting of the monoaminergic system to achieve successful analgesia for the treatment of chronic pain.

## Author Contributions

JM and LB performed the design of the structure of manuscript, analyzed the literature searching, wrote and revised several drafts of the manuscript. Also, they revised tables and contribute to perform the Figure 1. ML-T wrote the first part of the manuscript, revised the first draft of manuscript and elaborated the tables and the figure. EB analyzed the literature searching and revised the tables and the final draft of the manuscript.

## Conflict of Interest

The authors declare that the research was conducted in the absence of any commercial or financial relationships that could be construed as a potential conflict of interest.
